# Massive bleeding from gastric ulcer-induced splenic artery pseudoaneurysm successfully treated with transcatheter arterial embolization and surgery: a case report

**DOI:** 10.1186/s40792-022-01552-0

**Published:** 2022-10-11

**Authors:** Hidetoshi Shidahara, Nobuaki Fujikuni, Kazuaki Tanabe, Tomoyuki Abe, Keisuke Nishihara, Toshio Noriyuki, Masahiro Nakahara

**Affiliations:** 1 Department of Surgery, Onomichi General Hospital, Onomichi, Hiroshima Japan; 2 Department of Radiology, Onomichi General Hospital, Onomichi, Hiroshima Japan; 3 Department of Gastroenterological and Transplant Surgery, Graduate School of Biomedical and Health Sciences, Hiroshima University, Hiroshima, Japan; 4 Department of Gastroenterological Surgery, Hiroshima Prefectural Hospital, Hiroshima, Japan; 5 Department of Perioperative and Critical Care Management, Graduate School of Biomedical and Health Sciences, Hiroshima University, Hiroshima, Japan

**Keywords:** Peptic ulcer disease, Upper gastrointestinal bleeding, Splenic artery pseudoaneurysm, Transcatheter arterial embolization

## Abstract

**Background:**

Upper gastrointestinal bleeding (UGIB) is a routine medical emergency. The most common non-variceal cause is peptic ulcer disease, while a rare presentation is peptic ulcer-induced splenic artery pseudoaneurysm (SAP). Primary endoscopic treatment is generally attempted for UGIB; however, it sometimes fails when arterial etiology is present. In such cases, either transcatheter arterial embolization (TAE) or surgery is necessary, but the choice of treatment is controversial. We present a case that illustrates the utility of both approaches in a gastric ulcer-induced SAP.

**Case presentation:**

A 33-year-old male presented with hemorrhagic shock secondary to UGIB. The source of bleeding was identified as an SAP that was caused by a gastric ulcer. TAE enabled temporary bleeding control despite the patient’s poor overall condition and limited blood transfusion capability. However, rebleeding occurred soon after stabilization. Ultimately, we performed proximal gastrectomy and splenic artery ligation, and the patient survived.

**Conclusions:**

SAP is an uncommon occurrence, and angiographic information is important for correctly identifying the source of bleeding. The treatment for SAP bleeding is basically the same as for endoscopically unmanageable non-variceal UGIB, since TAE and surgery each have a different utility, depending on the situation. If surgery is performed, especially SA ligation and gastrectomy, it is important to consider the circulation of the spleen and residual stomach. Using TAE and laparotomy, we managed to save the life of the patient with massive hemorrhage under limited circumstances.

## Background

Upper gastrointestinal bleeding (UGIB) is a common medical emergency with a reported mortality of 2–10% [1]. Within UGIB, variceal and non-variceal etiologies are distinguished, as the management and patient outcomes differ. The most common cause of acute non-variceal UGIB is peptic ulcer disease (PUD) [2], with approximately 50% of all cases attributed to PUD [3]. One rare complication of PUD that can lead to massive UGIB is peptic ulcer-induced splenic artery pseudoaneurysm (SAP). So far, only a few cases have been reported.

Transcatheter arterial embolization (TAE) or surgical intervention are necessary for massive UGIBs that cannot be endoscopically controlled. The decision between surgery and TAE remains controversial, as these patients are often at high risk and are poor surgical candidates. TAE can save high-risk patients [4]; however, compared with surgery, it results in a higher rebleeding rate for uncontrolled peptic ulcer bleeding [5]. Nevertheless, the efficacy and safety of TAE in UGIB is lower than that in lower gastrointestinal bleeding or biliary hemorrhage, because the complex collateral circulation in the upper gastrointestinal tract can inhibit efficient embolization [6]. Here, we report the case of a patient with massive UGIB who required both interventions.

## Case presentation

**Fig. 1:**
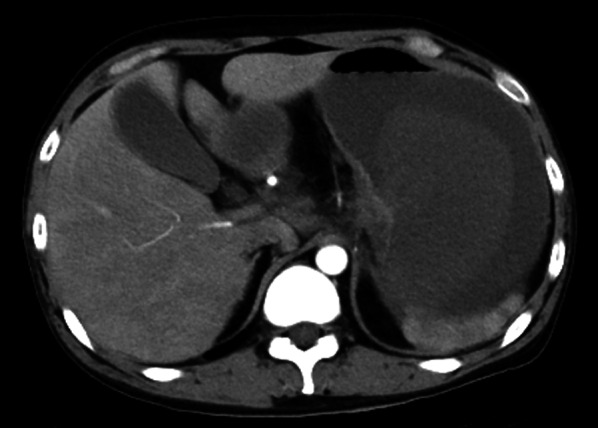
Dynamic computed tomography (CT) shows a massive, contrast-enhanced fluid collection in the stomach

**Fig. 2:**
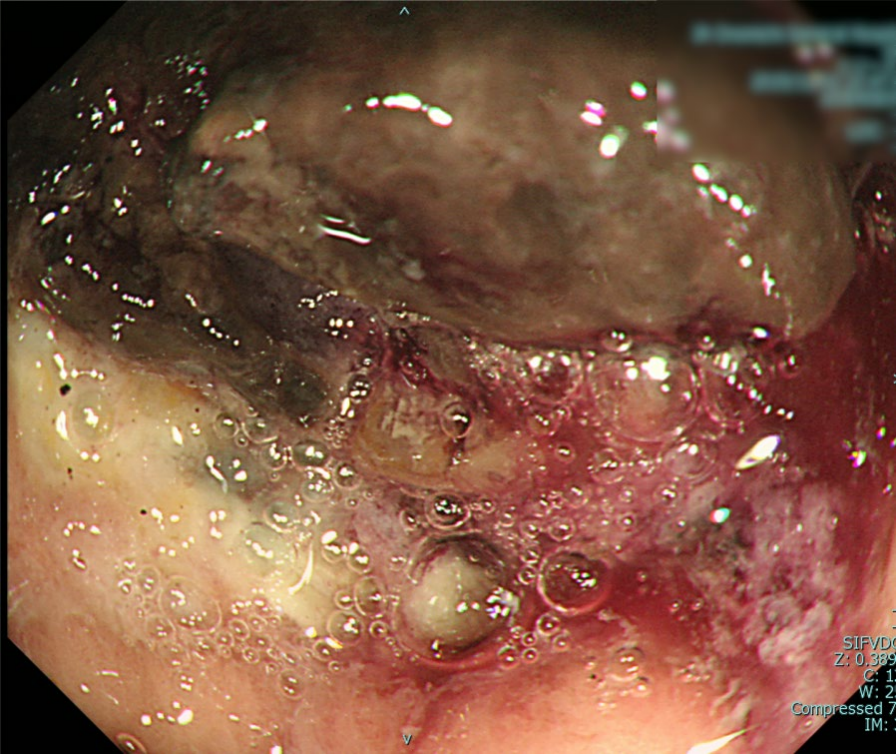
Upper gastrointestinal endoscopy shows the spontaneous hemostatic ulcer located in the upper posterior wall of the gastric body

An alcoholic 33-year-old male with a 2-week history of hematemesis presented to the hospital with pallor and impaired consciousness. The patient was hemodynamically unstable on arrival. On examination, his body temperature (BT) was 35.9 °C, heart rate was 120 beats per minute in sinus tachycardia, blood pressure was 84/34 mmHg, respiratory rate was 26 breaths per minute, and oxygen saturation was 100% on 10 L of oxygen. Laboratory results showed severe anemia (hemoglobin 3.7 g/dL) and poor pre-existing nutritional status (cholinesterase 81 U/L, albumin 1.9 g/dL). Dynamic computed tomography (CT) revealed a massive, enhanced fluid collection in the stomach (Fig. 1), while extravasation or free air were not observed. The patient underwent urgent upper gastrointestinal endoscopy. Massive fresh blood pooling was observed in the stomach and a gastric ulcer was located in the upper posterior wall of the gastric body (Fig. 2). No active bleeding was noted, and endoscopy was completed without clipping or cauterization.

Upon admission to the intensive care unit, his general condition improved with conservative treatment, including blood transfusion. On the second day after admission (on a holiday night), he experienced massive rebleeding, hematemesis, and sudden onset of hemorrhagic shock. Blood tests showed fatal anemia (hemoglobin 0.7 g/dL). The patient was managed urgently with massive blood transfusions and catecholamine administration. Cardiopulmonary resuscitation was required for several minutes. Rapid hemostatic intervention was necessary; however, the patient was in poor general condition, because of acidosis (pH 7.09) and hypothermia (BT 33.4 °C). Even worse, the preparation with transfusion was inadequate to conduct a laparotomy procedure. Therefore, emergency angiography and TAE were performed.

**Fig. 3:**
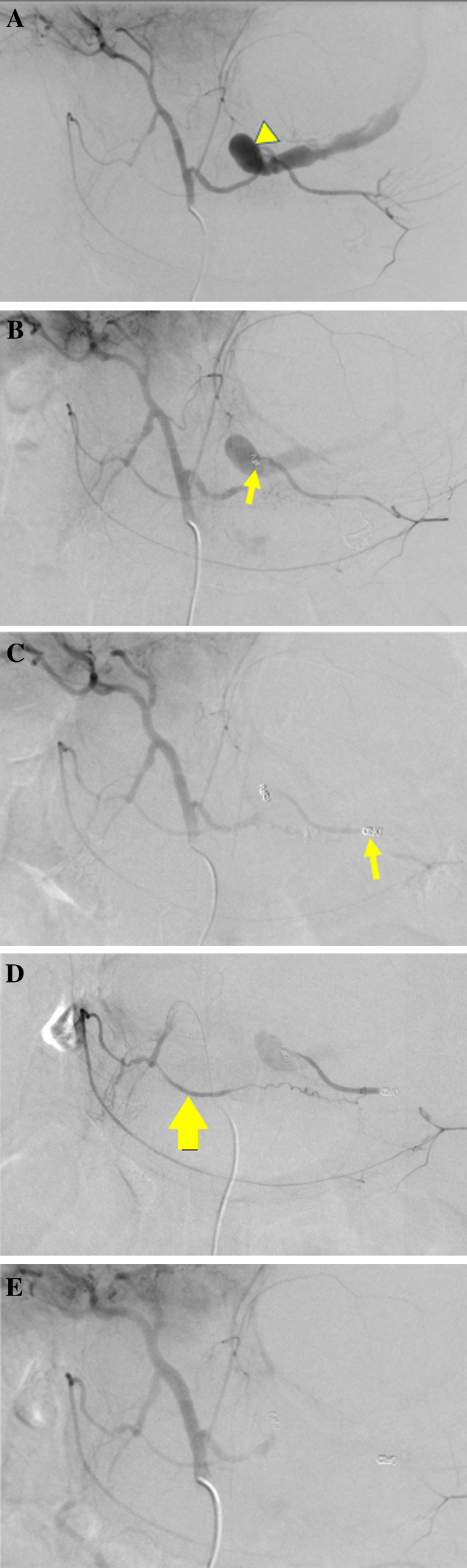
**A** Celiac angiography shows the splenic artery pseudoaneurysm (SAP) and extravasation from it (triangle). **B** Despite the proximal splenic artery (SA) being embolized with coils (arrow), extravasation persisted through the gastroduodenal artery and right gastroepiploic artery. **C**, **D** After the distal SA was embolized with coils (arrow in **C**)**,** extravasation from the SAP lessened but continued through the intrapancreatic arcade (arrow in **D**). **E** The arcade was embolized with a gelatin sponge, and then the extravasation dissipated

Celiac angiography revealed extravasation from a splenic artery pseudoaneurysm (SAP) (Fig. 3A). First, we aimed to isolate the SAP, but the catheter did not reach distal to the SAP from the celiac artery; there were vascular migrations from chronic pancreatitis and very limited utility of TAE. Reluctantly, we embolized the proximal splenic artery (SA) with a coil. The extravasation persisted through the gastroduodenal artery (GDA) and right gastroepiploic artery (RGEA) (Fig. 3B). The distal SA was then embolized with coils through the GDA and RGEA. Subsequently, the bleeding lessened (Fig. 3C), but it persisted through the intrapancreatic arcade (Fig. 3D). Coiling of the arcade was also difficult because of the vascular migrations. Finally, the arcade was embolized with a gelatin sponge and the extravasation dissipated (Fig. 3E).

**Fig. 4:**
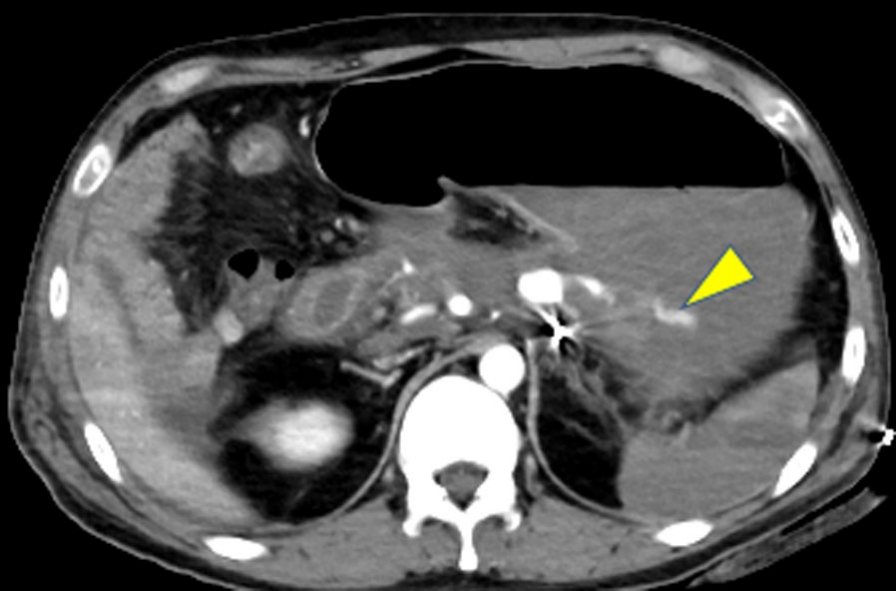
A second dynamic CT shows extravasation from the SAP into the stomach (triangle)

**Fig. 5:**
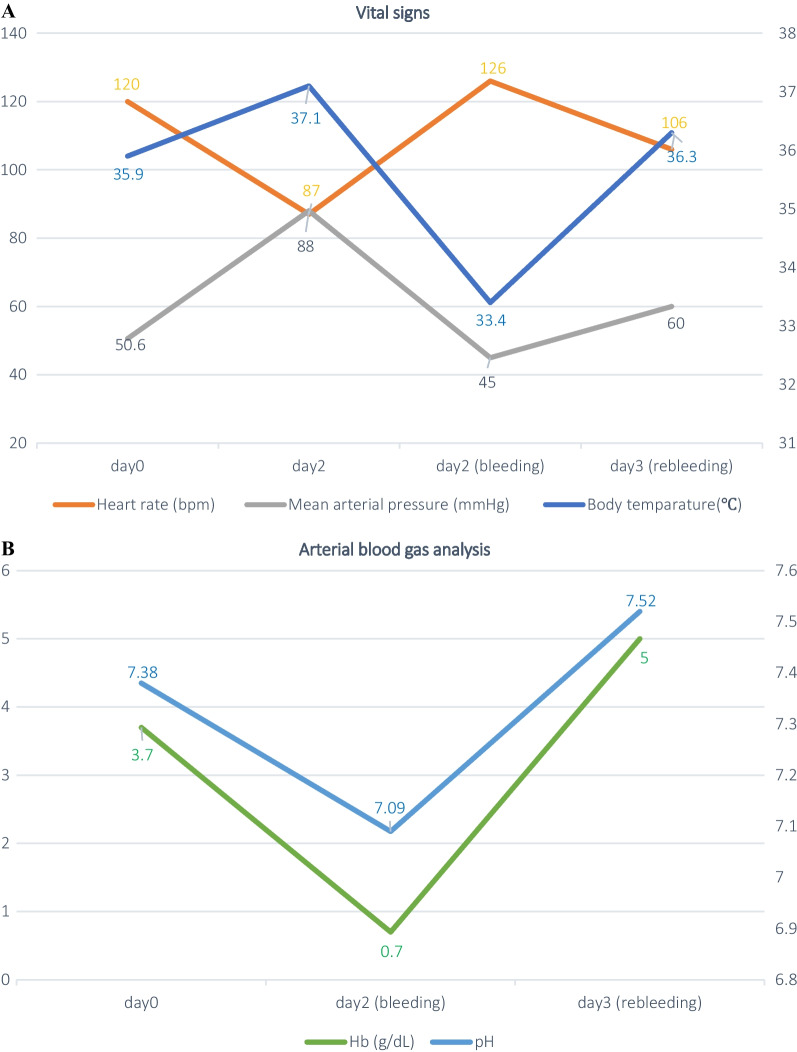
**A**, **B** Patient progress is summarized in chronological order. **A** Shows vital signs, and **B** shows laboratory data of arterial blood gas analysis

Although TAE accomplished a temporary cessation of bleeding, rebleeding occurred the following morning. Initial CT imaging was negative, but a second dynamic CT revealed extravasation from the SAP into the stomach (Fig. 4). The patient responded to fluids and blood transfusions, and vital signs were relatively stable (Fig. 5A). Acidosis and hypothermia were compensating, with pH 7.52 and BT 36.3℃ (Fig. 5B). During the daytime on a weekday, there were sufficient transfusions, clinical staff, and materials were ready for another surgical intervention. We decided to perform emergency laparotomy.

**Fig. 6:**
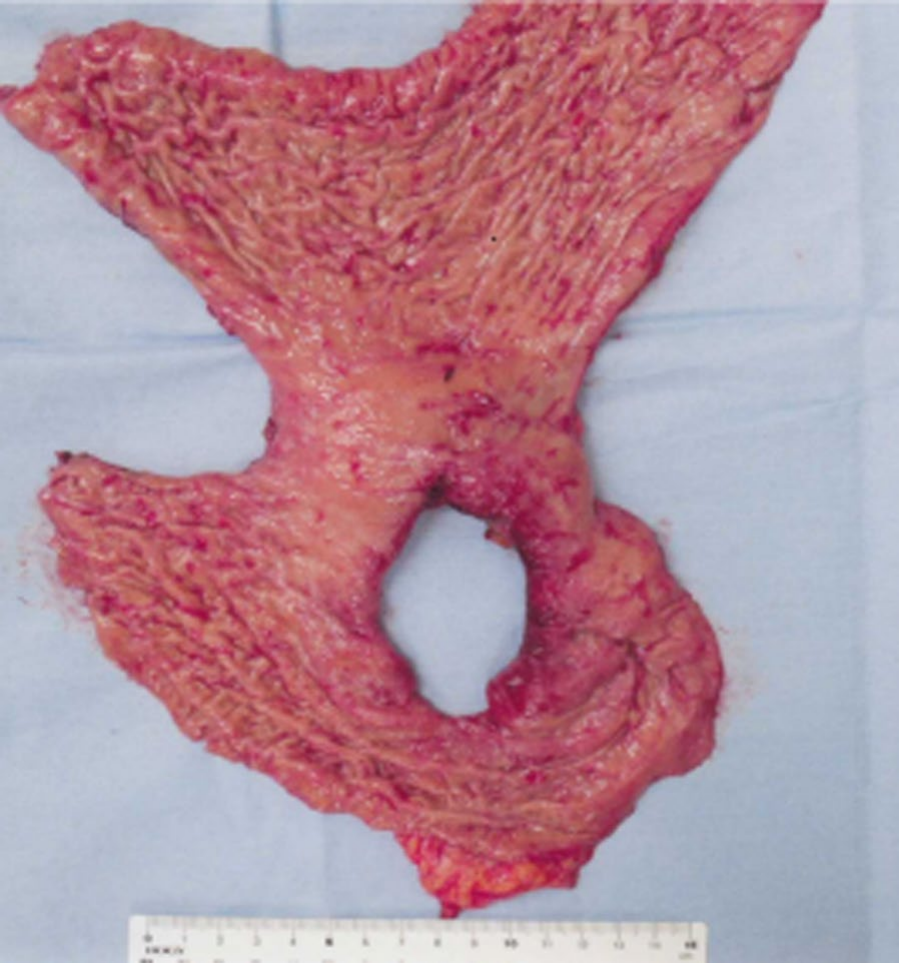
The resected specimen shows a perforated ulcer measuring 5.0 cm in diameter located on the posterior wall of the stomach body

Intraoperatively, the stomach was found to be filled with blood clots. A bleeding ulcer on the upper posterior wall of the stomach body was perforated, with a hole measuring 5.0 cm in diameter (Fig. 6). The SAP was identified as the source of bleeding. First, we ligated the SA proximal to the gastric ulcer perforation where the SAP was evident. At that point, hemostasis was obtained, and vital signs stabilized. Since the perforation was large, and simple suture or omentopexy was difficult, we decided to perform gastrectomy. Distal gastrectomy (DG) would have left the possibility of residual gastric necrosis from SA ligation. Therefore, we chose proximal gastrectomy (PG). The mobilization of gastric antrum was also not easy due to chronic pancreatitis. Therefore, we chose the double-tract method. The spleen and pancreas were preserved, because no visible color change was observed. The operation time was 196 min, and the intraoperative bleeding volume was 3000 mL.

The patient’s postoperative course was generally good. On postoperative day 7, contrast-enhanced CT showed partial splenic infarction; however, there was no intra-abdominal abscess or other abnormal findings. The patient was treated conservatively and discharged from the hospital on day 15 after surgery. The total transfusion during the hospitalization comprised 82 units of concentrated red blood cells, 50 units of fresh frozen plasma, and 40 units of platelets.

## Discussion

PUD is the main cause of non-variceal UGIB [3], with duodenal ulcers more likely to experience bleeding than the gastric ulcers [7]. However, SAP is a rare occurrence. In a large series from the Mayo Clinic, only 10 SAPs were identified in patients over 18 years of age. The SAP is most often caused by pancreatitis (52%) or trauma (29%) and rarely by PUD (2%) [8].

To the best of our knowledge, seven cases of PUD-induced SAP have been reported in the past 14 years. Including our case, Table 1 summarizes the characteristics of patients, treatment, and outcomes [9–15] (Table 1). The most common symptoms were abdominal pain and hematemesis (5/8 cases, 62.5% each), followed by melena (4/8 cases, 50%) and neurological symptoms (3/8 cases, 37.5%). As major bleeding occurred, the patients were highly anemic and vital signs were often unstable. On initial endoscopy, the PUD was often located on the posterior wall of the stomach (6/8 cases, 75%), followed by the duodenum (1/8 case, 12.5%). When PUD is present in the posterior wall of the stomach or duodenum, UGIB by arterial perforation, including SAP bleeding, should be considered in the differential diagnosis. An earlier review mentioned that SAP was most commonly diagnosed by angiography (52%) followed by CT (36%) [8]. In two cases, including ours, a repeat CT scan correctly identified the source of bleeding [13]. Unfortunately, one case was misdiagnosed as a cystic tumor on the initial non-contrast CT and biopsied [14]. The lesson from the cases above is that angiographic information is essential in UGIB.

**Table 1:** Reported cases of peptic ulcer disease-induced splenic artery pseudoaneurysm

Author	Year (report)	Age	Sex	Comorbidity, background	Symptoms	Hemodynamics	Hemoglobin (g/dL)	Location of PUD	Primary treatment	Rebleed	Surgical procedure	For SAP	Outcome	Complications
Pasumarthy [9]	2009	55	M	HT, HL, DM, coronary artery disease	Abdominal pain, hematemesis, melena	Unstable	8.1	Stomach (within 2 cm to EGJ)	TAE	+	Total gastrectomy + splenectomy	NA	Saved	NA
Syed [10]	2014	77	F	None	Abdominal pain, hematemesis	Stable	7.7	Stomach (posterior wall)	TAE	−	Partial gastrectomy + DP + splenectomy	Resected	Saved	None
Varshney [11]	2014	38	M	NA	Abdominal pain, hematemesis, melena	NA	4	Stomach (posterior wall)	laparotomy	−	Primary repair + splenectomy	NA	Saved	None
Sawicki [12]	2015	57	M	None	Abdominal pain, syncope	Unstable	6.7	Stomach (posterior wall)	laparotomy	−	Primary repair only	Bipolar ligated	Saved	Acute respiratory distress syndrome
Cho [13]	2018	61	M	Homeless person	Dizziness, melena	Stable	2.7	Stomach (posterior wall)	TAE × 2 times	+	Gastrectomy (details unknown)	NA	Saved	None
Menaria [14]	2019	57	F	None	Abdominal pain, hematemesis	Unstable	6.4	Duodenum (the fourth part)	TAE	−	–	–	Saved	Splenic infarction
Nakata [15]	2022	72	F	Atrial fibrillation, HT	Melena	Unstable	4	Stomach (posterior wall)	REBOA	−	Distal gastrectomy	Resumed with suture	Saved	None
Our case	2022	33	M	None	Hematemesis, disturbance of consciousness	Unstable	3.7	Stomach (posterior wall)	TAE	+	Proximal gastrectomy	Ligated	Saved	Partial splenic infarction

*DM* diabetes mellitus, *DP* distal pancreatectomy, *EGJ* Esophagogastric junction, *F* female, *HL* hyperlipidemia, *HT* hypertension, *M* male, *REBOA* resuscitative endovascular balloon occlusion of the aorta, *PUD* peptic ulcer disease, *TAE* transcatheter arterial embolization

*NA* not available

The treatment of SAP bleeding is fundamentally the same as for endoscopically unmanageable non-variceal UGIB; to date, various methods have been reported. Of the eight published cases mentioned above, primary treatment often included TAE (5/8 cases, 62.5%), with rebleeding occurring in approximately half of the TAE group. According to one previous report, the rebleeding rate was higher in the TAE group than in the surgery group (HR 2.48, 95% CI 1.33–4.72) [5]. On the other hand, considering all secondary hemostatic interventions, both groups achieved approximately 87% durable hemostasis. Rebleeding within 3 days (OR 3.7, *p* = 0.042), and corticosteroid use (OR 5.2, *p* = 0.017) had significant negative impacts on survival [16].

Laparotomy was performed in most cases (7/8 cases, 87.5%). In two cases, surgery was performed at the start; the patients survived without rebleeding. In three post-TAE rebleeding cases, including ours, surgery was chosen as the secondary intervention and achieved good results. In one case without rebleeding, early laparotomy was performed for curative purposes. The resuscitative endovascular balloon occlusion of the aorta (REBOA) technique has been reported to be useful for decreasing SAP bleeding volume [15]. Also, resuscitative thoracotomy with aortic cross-clamp (RTACC) is indicated for imminent cardiac arrest. In every case, the procedure was designed to provide rapid control of blood loss, leading to reliable hemostasis.

As regards complications, in one case, splenic infarction occurred after coil embolization of the splenic artery [14]. In other cases, splenic blood flow was maintained despite splenic artery ligation or embolization; collateral blood circulation from the inferior phrenic artery or omentum is thought to be involved. In our case, collateral circulation was expected from the intraoperative findings, so splenectomy was avoided at that time. As a result, the only complication was partial splenic infarction, which was followed conservatively. If a splenic abscess forms postoperatively, splenectomy should be considered.

There is another risk, that of residual gastric necrosis, when DG and splenic artery ligation are performed simultaneously, which Nakata et al. mitigated by using open abdominal postoperative management [15]. In our case, PG and the SA ligation were performed, and residual stomach circulation was preserved through the RGEA and right gastric artery. To avoid residual gastric necrosis, PG or total gastrectomy was found to be safer than DG, because splenic artery ligation or embolization is always necessary in SAP bleeding.

Finally, multidisciplinary and rapid treatment is required to save lives in case of SAP bleeding. Although in limited circumstances, we managed to temporarily control the critical bleeding with TAE. When rebleeding occurred, we considered that blood flow from the intrapancreatic arcade, which had been embolized with a gelatin sponge, had resumed. Therefore, we concluded that no further IVR would be effective; we decided to perform surgery. Retrospectively considering our case, the best way to respond to shock requiring cardiopulmonary resuscitation is REBOA or RTACC; however, each facility’s background, medical resources, timeframe, and other conditions are different. Establishing an emergency medical care system that makes these interventions possible is a future challenge for regional hospitals.

## Conclusions

This is a case report of SAP bleeding caused by a gastric ulcer. Using TAE and laparotomy, we managed to save the life of a patient with massive hemorrhage under limited treatment circumstances. When endoscopic treatment fails or is difficult, TAE or surgery are necessary. TAE can save poor surgical candidates like our patient; however, the rebleeding rate is higher than with surgery. If surgery is performed for SAP, especially SA ligation and gastrectomy, it is important to consider the vascularization of the spleen and residual stomach. In fatal circumstances, REBOA or RTACC should be considered for reducing blood loss. The establishment of an emergency medical care system is a future issue.

## Data Availability

Data sharing is not applicable to this article, as no datasets were generated or analyzed during the current study.

## Abbreviations

BT: Body temperature
CT: Computed tomography
DG: Distal gastrectomy
GDA: Gastroduodenal artery
PG: Proximal gastrectomy
PUD: Peptic ulcer disease
REBOA: Resuscitative endovascular balloon occlusion of the aorta
RGEA: Right gastroepiploic artery
RTACC: Resuscitative thoracotomy with aortic cross-clamp
SA: Splenic artery
SAP: Splenic artery pseudoaneurysm
TAE: Transcatheter arterial embolization
UGIB: Upper gastrointestinal bleeding
